# How do plant roots overcome physical barriers?

**DOI:** 10.1093/jxb/erac238

**Published:** 2022-08-11

**Authors:** Daiyan Li, Zhongtao Jia

**Affiliations:** Triticeae Research Institute, Sichuan Agricultural University, 611130 Chengdu, China; Key Laboratory of Plant-Soil Interactions, MOE, College of Resources and Environmental Sciences, National Academy of Agriculture Green Development, China Agricultural University, 100193 Beijing, China; Molecular Plant Nutrition, Department of Physiology and Cell Biology, Leibniz Institute of Plant Genetics and Crop Plant Research, D-06466 Gatersleben, Germany

**Keywords:** Auxin, calcium signaling, ethylene, mechanical impedance, natural variation, reactive oxygen species, root penetrability

## Abstract

This article comments on:

**Bello-Bello E, Rico-Chambrón TY, Ortiz Ramírez LA, Rellán Álvarez R, Herrera Estrella L.** 2022. ROOT PENETRATION INDEX 3, a major quantitative trait locus associated with root system penetrability in Arabidopsis. Journal of Experimental Botany **73**, 4716–4732.

**Soil drying and compaction increase mechanical impedance, a common problem in agricultural soils that adversely impacts crop productivity. Plant roots with improved capacity to penetrate hard soil layers can more efficiently explore nutrients and water available in deeper root zones. In this issue, Bello-Bello et al. (2022a) demonstrated that auxin dynamics have a critical role in facilitating root penetration into hard barriers, and mapped a large-effect quantitative trait locus (QTL), *ROOT PENETRATION INDEX 3*, associated with primary root penetrability in *Arabidopsis thaliana*. These findings give new insights into the regulatory role of auxin signaling in root penetrability and shed light on related natural genetic variation for this important trait.**

## Growth responses to mechanical obstacles

When growing through the soil profile, plant roots encounter many physical obstacles in their path and must adapt their growth behavior through complex interactions of differently timed responses. Depending on the root growth pressure and physicochemical properties of the surroundings in which they are growing, plants interchangeably employ obstacle-avoidance and obstacle-overcoming strategies to adapt to compaceted soils. Upon touching a barrier, the root tip rapidly bends away from the obstacle, a response called thigmotropism, followed by a second bending along the gravity vector mediated by gravitropism, resulting in a step-like growth pattern (Massa and Gilroy, 2003). Both of these consecutive bending responses rely on asymmetrical cell expansion in the root elongation zone (Lee *et al.*, 2020). Combined thigmotropism and gravitropism are thought to enable roots to grow downwards and penetrate physical barriers (Lee *et al.*, 2020).

To study the impact of a continuous mechanical constraint on root growth, Bello-Bello *et al.* (2022a) established a two-phase agar system composed of a thin upper and a thick lower layer that contains agar (a gelling agent) at different concentrations. Using this growth system, the authors performed detailed analyses on roots which were undisturbed, or were able or not to penetrate the lower layer, in order to probe anatomical and morphological changes in response to mechanical stress. Their results showed that in comparison with undisturbed Arabidopsis plants, seedlings that failed to penetrate the agar layers (at low and high mechanical impedance) exhibited a shorter primary root and elongation zone but comparable meristem size. The activity of the quiescent center and cell division, assessed using *QC46::GUS* and *CYCB1;1::GFP* reporters, respectively, were not remarkably different between roots among three different penetration stages. These results are in line with previous findings that inhibition of cell elongation is the major determinant of reduced root length in response to physical impedance (Okamoto *et al.*, 2008). Interestingly, although both cell length and root elongation were also reduced in roots that could penetrate the hard agar, the extent of inhibition was less drastic than in impeded roots, and the leaf area of shoots was significantly increased. These data overall reinforce the agronomical relevance of the root system penetrability, in line with a recent report that deep soil penetrability improves crop growth in compacted soils (Schneider *et al.*, 2021).

While inter- and intraspecific variation has been documented for root penetrability in several plant species (Lynch *et al.*, 2022), how existing natural genetic variation effectively determines root penetration in response to mechanical constraints remains unclear. Bello-Bello *et al.* (2022a) found substantial variation for primary root penetrability in a panel of *A. thaliana* accessions and used a recombinant inbred line (RIL) population (Ler-0×Sha) to uncover a major-effect QTL associated with this root trait. However, it remains unclear whether the observed genetic diversity was due to differences in thigmotropism, gravitropism, or both. Importantly, the root penetrating ability of the IL321 line with introgression of a genomic fragment of Sha to Ler-0 was significantly decreased compared with its parental line Ler-0, suggesting that the Sha allele negatively contributes to root penetrability. Bioinformatics analysis prioritized a number of candidate genes involved in hormonal pathways related to root soil mechanical response. As such, in future, functional characterization of these candidate gene(s) and mining causal variation underlying the identified QTLs will be of utmost importance. This will not only extend our mechanistic understanding of root mechanical sensing, but will also shed light on ecological adaptation of plants to soil impedance.

## Auxin steers roots to penetrate hard barriers

Auxin is one of the major phytohormones that regulates root development and plastic responses to environmental cues (Jia *et al.*, 2022). Recent studies have shown that auxin signaling and particularly PIN-mediated auxin transport facilitates early mechanical impedance responses; that is, obstacle avoidance (Lee *et al.*, 2020; Jacobsen *et al.*, 2021). To visualize auxin responses triggered during root penetration, Bello-Bello *et al.* (2022a) analyzed the expression of the auxin-responsive marker *DR5::GFP* in reporter plants grown under low and high mechanical stress. They found that mechanical constraints significantly increased auxin responses in specific root tissues including the quiescent center, columella cells, and epidermal cells of the elongation zone. Furthermore, the authors showed that mechanical impedance strongly decreased the abundance of PIN2 in the epidermal and cortical cells, and of PIN3 and PIN7 in the stele, suggesting that the presence of a continuous mechanical stress promotes auxin redistribution as a critical step for enhancing root penetrability. This conclusion was further consolidated by demonstrating that the auxin transporter mutants *pin2* and *aux1* completely lost their ability to penetrate the hard agar layer, while exogenous supply of auxin not only partially rescued root penetration of the two mutants but also increased the penetrability of wild-type roots. In summary, these findings support that auxin transport and signaling steer root growth and development to facilitate penetration of a hard barrier.

Auxin dynamics within the root tissues are coordinated by an integrated action of *de novo* biosynthesis, metabolism, and intra- and intercellular transport processes (Casanova-Sáez *et al*., 2021). With regard to root penetrability, it still remains unclear whether local auxin biosynthesis is required and how downstream auxin signaling events regulate this adaptive response. Interestingly, Bello-Bello *et al.* (2022a) found that natural accessions with contrasting root penetrability also show distinctive sensitivity to exogenous auxin, suggesting a link between root penetration capacity and variation in auxin response. Whether these differential responses to auxin relate to variation in components involved in auxin biosynthesis, transport, or signaling merits further investigation. Notably, recent work uncovered that natural allelic variants of *YUC8* and *EXOCYST70A3*, modulators of auxin biosynthesis and transport, respectively, determine natural variation for cell elongation and variable root adaptation to low nitrogen (N) and soil drought (Ogura *et al.*, 2019; Jia *et al.*, 2021). Although the adverse impact of shallow rooting caused by soil compaction on root foraging for nutrient and water has been proposed, it remains unknown to what extent root penetrability impacts on the root system adaptive response to low N and soil drought. In this regard, it will be interesting to examine their roles and related allelic variants in the context of soil compaction, which may shed light on how natural selection of these different growth constraints converged evolutionarily on auxin signaling pathways.

## What else is beyond auxin?

Several other signals such as calcium (Ca^2+^), reactive oxygen species (ROS), extracellular ATP (eATP), and pH have been implicated in root mechanosensing and mechanotransduction (Bello-Bello *et al.*, 2022b). Mechanostimulation triggers rapid cytoplasmic Ca^2+^ transients via activity of the the plasma membrane-localized ion channels MCA1 and PIEZO1 (Nakagawa *et al.*, 2007; Mousavi *et al.*, 2021), which in turn activates downstream ROS production and extracellular pH changes (Monshausen *et al*., 2009). In addition, mechanical stimuli-induced Ca^2+^ responses are regulated by the receptor-like kinase FERONIA that is proposed as a co-receptor to monitor cell wall status (Shih *et al.*, 2014). Downstream of these early signaling events, plant hormones such as ethylene, auxin, and abscisic acid regulate root penetration (Bello-Bello *et al.*, 2022b). For example, ethylene has been reported as a warning signal that stops root elongation in response to compact soil (Pandey *et al.*, 2021). Consequently, ethylene signaling mutants (*ein2*, *ein3*, and *ctr1*) or insensitive mutants (*aux1* and *pin2*) either grow longer or are less inhibited when encountering the physical barrier, corroborating the model that ethylene-induced auxin biosynthesis and redistribution is a driving force for root growth inhibition under mechanical stress (Okamoto *et al*., 2008, 2021; Santisree *et al.*, 2011). There is evidence that ROS and ethylene signaling pathways interact in a feedback regulatory loop (Xia *et al.*, 2015). Mutual activation of ROS production and Ca^2+^ signaling has been observed, in that NADPH oxidase-produced ROS enables activation of Ca^2+^ channels and the increase in Ca^2+^ in turn further activates the NADPH oxidase activity (Gilroy *et al.*, 2016). It is plausible that the concerted actions of these signaling pathways function interdependently to determine the root mechanical response. Thus, some of the outstanding questions for future research are to explore further interactions of ROS, Ca^2+^, ethylene, and auxin during root penetration of hard barriers.
